# Supplementary material for: In-depth analysis of transcriptomes in ovarian cortical follicles from children and adults reveals interfollicular heterogeneity
Source: Nat Commun. 2024 Aug 21;15:6989. doi: 10.1038/s41467-024-51185-0 (PMC11339373; doi:10.1038/s41467-024-51185-0)
Supplement: Supplementary file 1 — Supplementary Information [file 41467_2024_51185_MOESM1_ESM.pdf]

In-depth analysis of transcriptomes in ovarian cortical follicles from children and adults reveals interfollicular heterogeneity

Ilmatar Rooda*, Jasmin Hassan, Jie Hao, Magdalena Wagner, Elisabeth Moussaud-Lamodière, Kersti Jääger, Marjut Otala, Katri Knuus, Cecilia Lindskog, Kiriaki Papaikonomou, Sebastian Gidlöf, Cecilia Langenskiöld, Hartmut Vogt, Per Frisk, Johan Malmros, Timo Tuuri, Andres Salumets, Kirsi Jahnukainen, Agne Velthut-Meikas,# , Pauliina Damdimopoulou#,*

Supplementary Results

Supplementary Figure 1 Page 2

Supplementary Figure 2 Page 4

Supplementary Figure 3 Page 6

Supplementary Figure 4 Page 8

Supplementary Figure 5 Page 10

Supplementary Figure 6 Page 11

Supplementary Figure 7 Page 13

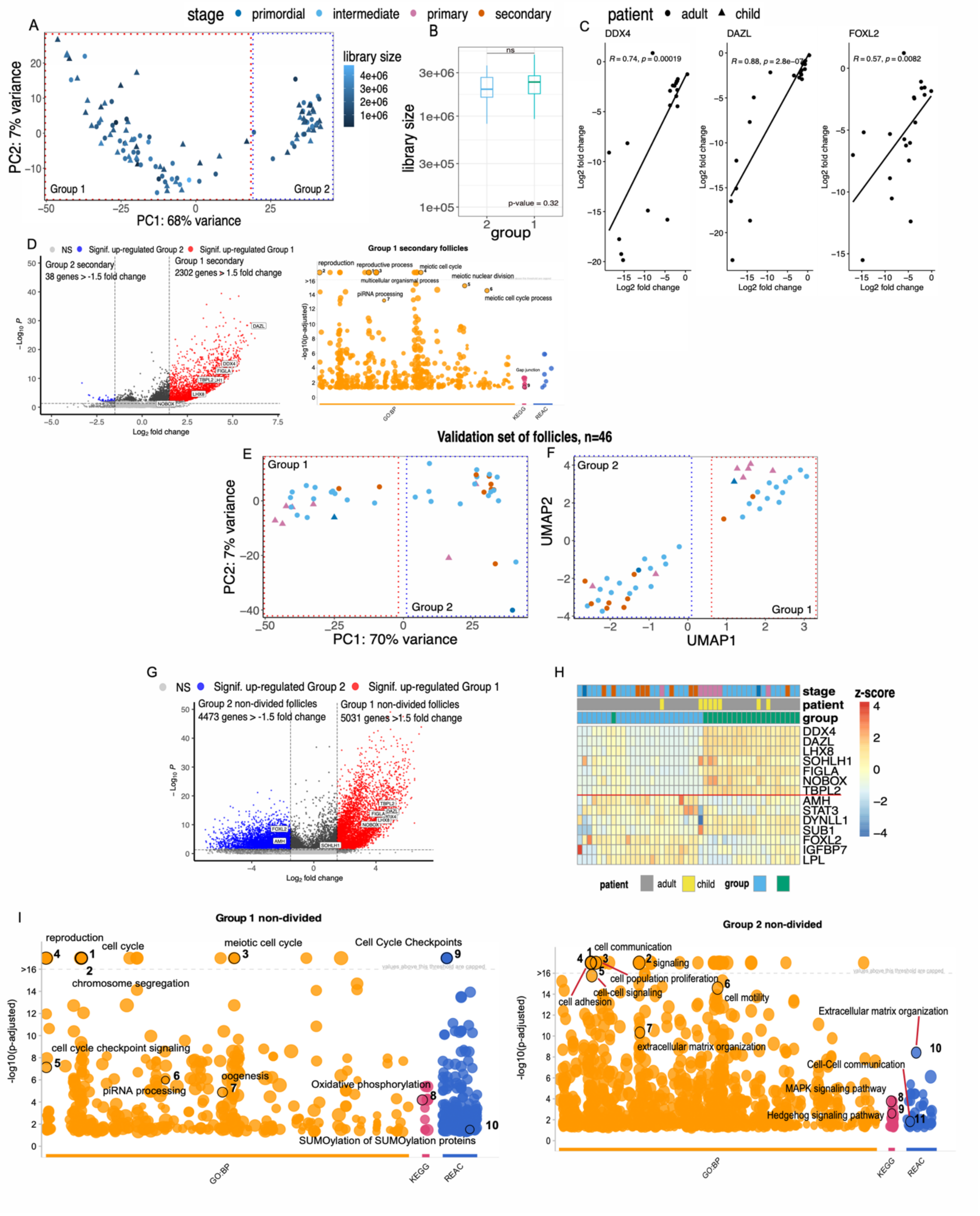

Supplementary Figure 1. Validation of Group 1 and Group 2 follicles.
A-B) Library size did not account for the division of follicles into two groups (n=109). In box plots, the center line represents the median, the hinges correspond to the first and third quartiles (interquartile range), and the whiskers extend to 1.5 times the interquartile range from the hinges.
C) qPCR analysis of the two halves of divided follicles (n=20) displayed a significant Pearson correlation, suggesting that splitting creates two comparable halves.

D) Volcano plot showed marked upregulation of genes in Group 1 secondary follicles compared (n=10) to Group 2 (n=16). Top enriched Gene Ontologies (GO), KEGG, and Reactome pathways in Group 1 secondary follicles suggested enrichment of reproductive processes and meiotic cell cycle.
E-F) A validation set of 46 viable follicles of good morphology were isolated from child and adult subjects following the main study protocol and subjected to Smart-seq2 sequencing without splitting. Principal component analysis (PCA) and uniform manifold approximation and projection for dimension (UMAP) visualization revealed a similar division of the follicles into two groups, as observed in the main study follicles.
G-H) Consistent with the main study results, a volcano plot showed that Group 1 follicles were characterized by marked upregulation of genes compared to Group 2, with the top DEGs including multiple known oocyte and granulosa cell markers.
I) The top enriched biological processes (yellow dots), KEGG (red dots), and Reactome (blue dots) pathways suggested reproduction and meiotic processes as the most significant terms in Group 1 follicles, and cell signaling and extracellular matrix as most significant terms in Group 2 follicles. The size of the dot corresponds to the size of the corresponding term, and terms on the X-axis from the same GO subtree are located closer to each other. NS- non-significant.

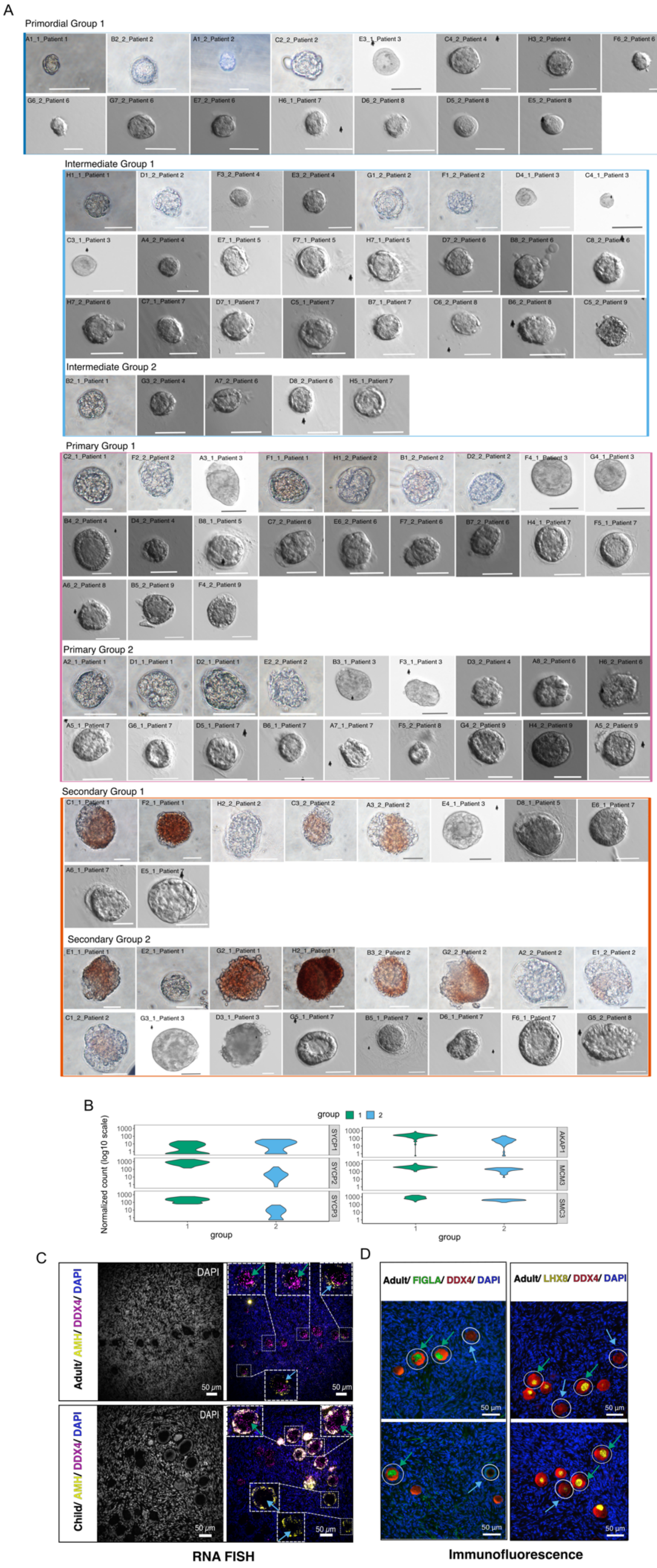
A
Primordial Group 1
Intermediate Group 1
Intermediate Group 2
Primary Group 1
Primary Group 2
Secondary Group 1
Secondary Group 2
B
group
Normalized count (log10 scale)
SYCP1
SYCP2
SYCP3
AKAP1
MCM3
SMC3
C
Adult/ AMH/ DDX4/ DAPI
Child/ AMH/ DDX4/ DAPI
DAPI
50 µm
RNA FISH
D
Adult/ FIGLA/ DDX4/ DAPI
Adult/ LHX8/ DDX4/ DAPI
Immunofluorescence

Supplementary Figure 2. Morphology of Group 1 and Group 2 follicles

A) Microscopic images of the follicles in the study (n=109), arranged by morphologically determined developmental stages. Within each stage, follicles are further categorized into Group 1 and Group 2 follicles based on their transcriptomic profiles. Scale 50 µm. Each individual follicle is labelled according to the same principle as in the count matrix, which is available under accession number GSE241984.

B) Selected genes involved in meiosis display expression in both groups of follicles (Group 1 n=70 and Group 2 n= 39).

C) mRNA hybridization targeting *AMH* and *DDX4* transcripts in child (n=1) and adult (n=1) ovarian cortex revealed follicles with varying transcript levels. Inserts spotlight potential Group 1 follicles (higher *DDX4*, lower *AMH*, indicated by green arrows) and Group 2 follicles (lower *DDX4*, higher *AMH*, indicated by blue arrows).

D) Immunofluorescence staining of adult ovarian cortex (n=2) indicates follicles with varying nuclear expression of crucial oocyte transcription factors FIGLA and LHX8 at the protein level. Blue arrows point to presumptive Group 2 follicles, and green arrows to Group 1 follicles. Only follicles with visible nuclei are annotated.

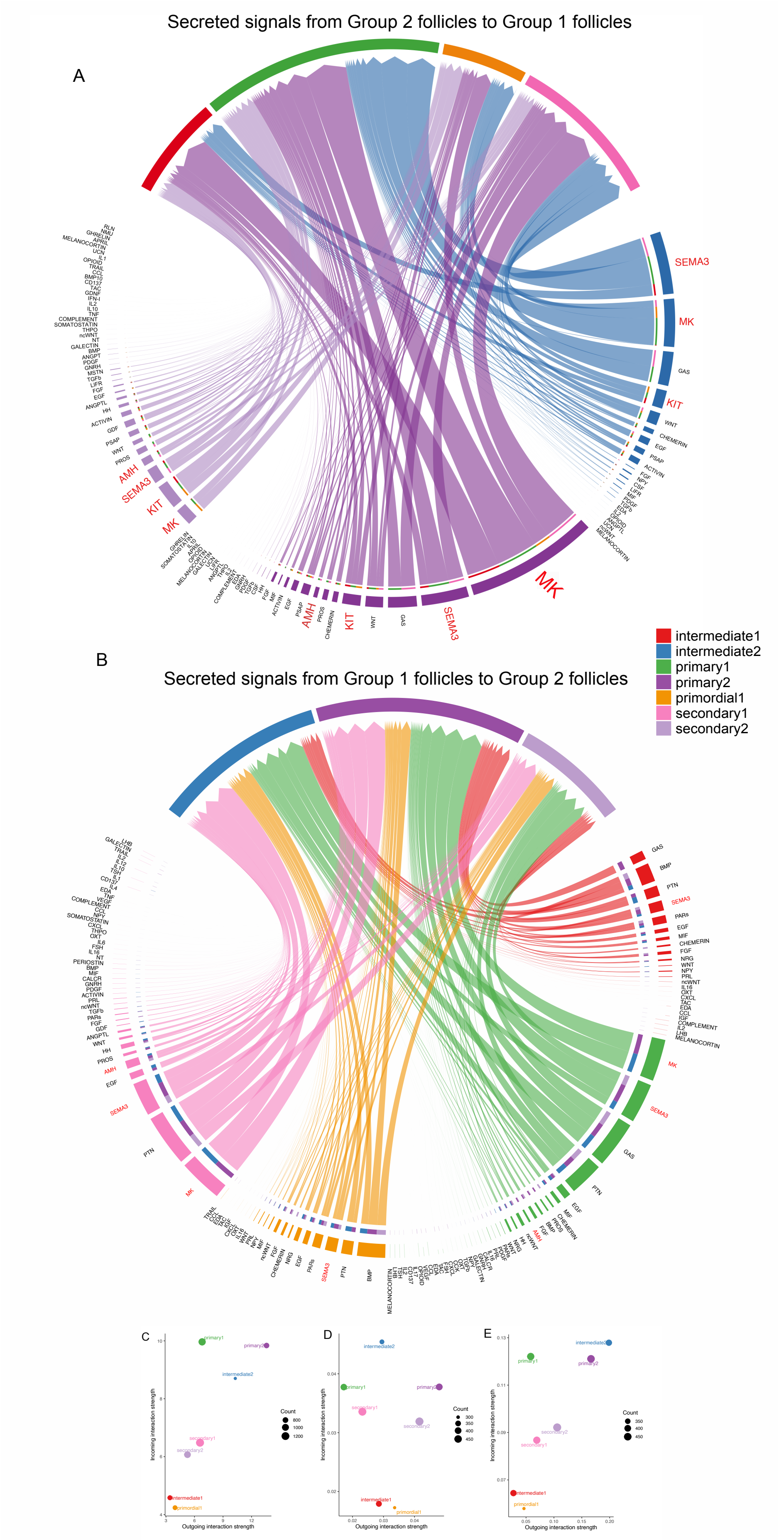
Secreted signals from Group 2 follicles to Group 1 follicles
A
SEMA3
MK
KIT
AMH
B
Secreted signals from Group 1 follicles to Group 2 follicles
intermediate1
intermediate2
primary1
primary2
primordial1
secondary1
secondary2
C
D
E
Incoming interaction strength
Outgoing interaction strength
Count

Supplementary Figure 3. Group 2 follicles demonstrate potential communication with Group 1 follicles via secreted signals

A) Chord plot visualizing signaling interactions between Group 2 (sources, n=39) and Group 1 (targets, n=70) follicles. The chords illustrate the signaling interactions from Group 2 to Group 1 follicles, with chord thickness representing the interaction strength. Follicular stages are depicted by outer circle colors (outer thicker circle for sending follicle stage; inner thinner circle for receiving follicle stage), and each segment's size indicates the relative prominence of the follicle type in signaling interactions. Predominant secreted signals from Group 2 follicles across all stages included neuronal growth factors MK (midkine) and SEMA-3 (semaphorin-3), along with recognized follicle-derived growth factors KIT (KIT proto-oncogene, receptor tyrosine kinase) and AMH (anti-Müllerian hormone).

B) Chord plot visualizing signaling interactions between Group 1 (sources) and Group 2 (targets) follicles. Predominant secreted signals from Group 1 follicles across all stages include similar signals like Group 2 follicles (MK, SEMA-3 and AMH).

C-E) Scatter plot overview of cell-to-cell communications among follicles using different normalization methods displays same patterns with highest outgoing interactions strengths in Group 2 follicles. C) CellChat normalization; D) Deseq2 normalization and E) EdgeR normalization.

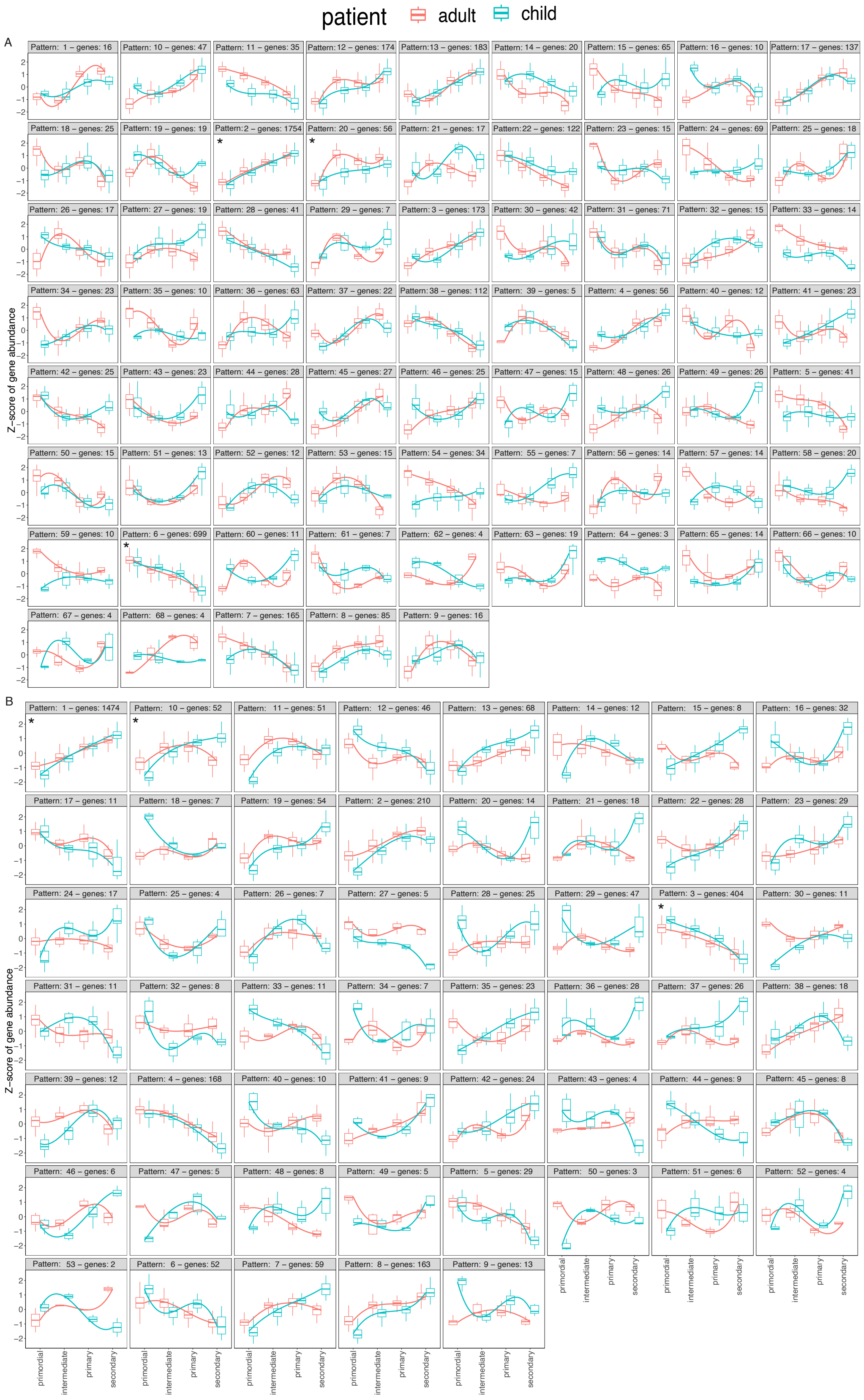

C

Adult down-sampling (n=6)	Set 1 DEGs	Set 2 DEGs	Set 3 DEGs	Set 4 DEGs	Set 5 DEGs	Range	SD	Full set follicles DEGs	Overlap %
Primordial to intermediate	956	1252	978	1327	1081	956-1327	164,96	900	86
Primordial to primary	2185	2041	1596	1665	1431	1431-2158	316,608	2336	80
Primordial to secondary	3854	4398	3438	3877	3598	3438-4398	364,984	3812	96
Intermediate to primary	589	1062	649	1167	1109	589-1167	273,761	527	83
Primary to secondary	444	471	410	613	530	410-613	79,9081	290	91

Child down-sampling (n=4)	Set 1 DEGs	Set 2 DEGs	Set 3 DEGs	Set 4 DEGs	Set 5 DEGs	Range	SD	Full set follicles DEGs	Overlap %
Primordial to intermediate	337	1526	869	569	1464	337-1526	529,957	1151	51
Primordial to primary	375	2169	1643	1749	1557	375-2169	670,611	1552	76
Primordial to secondary	961	2688	1658	808	2212	808-2688	802,473	2563	71
Intermediate to primary	218	635	346	280	823	218-823	258,047	168	20
Primary to secondary	273	367	191	194	651	191-651	190,618	127	56

Supplementary Figure 4. Gene expression patterns during follicle development
DEGs were subjected to gene expression patterns analysis through follicle development, from primordial to secondary stage, using the degPatterns package. Differentially expressed genes (DEGs) were utilized as input to perform pair-wise gene expression calculation, grouping genes into similar expression patterns. The y-axis in the figure is centered on the mean and scaled to the standard deviation by each gene.
A) Gene expression patterns derived from adult DEGs (red boxes). Corresponding child genes are shown in blue for comparison. In box plots, the center line represents the median, the hinges correspond to the first and third quartiles (interquartile range), and the whiskers extend to 1.5 times the interquartile range from the hinges.
B) Gene expression patterns derived from child DEGs (blue boxes). Corresponding child genes are shown in blue for comparison. In box plots, the center line represents the median, the hinges correspond to the first and third quartiles (interquartile range), and the whiskers extend to 1.5 times the interquartile range from the hinges.
FDR filter for DEGs <0.05. Patterns marked with an asterisk were utilized in GO and pathway enrichment analyses, as presented in main Figure 3.
C) Due to the unequal distribution of follicles between different follicle stages down-sampling to the smallest number of follicles was tested (adult n=6 and child n=4). Adult samples showed that a full set of Group 1 follicles captures 80-96% of the same genes as 5 down-sampling sets of follicles do. In child samples due to the smaller set size (n=4 follicles) higher variability was detected in down-sampled sets and the full Group 1 follicle set captured 20-76% of DEGs. Therefore, for downstream analysis full follicle set results were used. All DEGs from different comparisons are added to the Source Data.

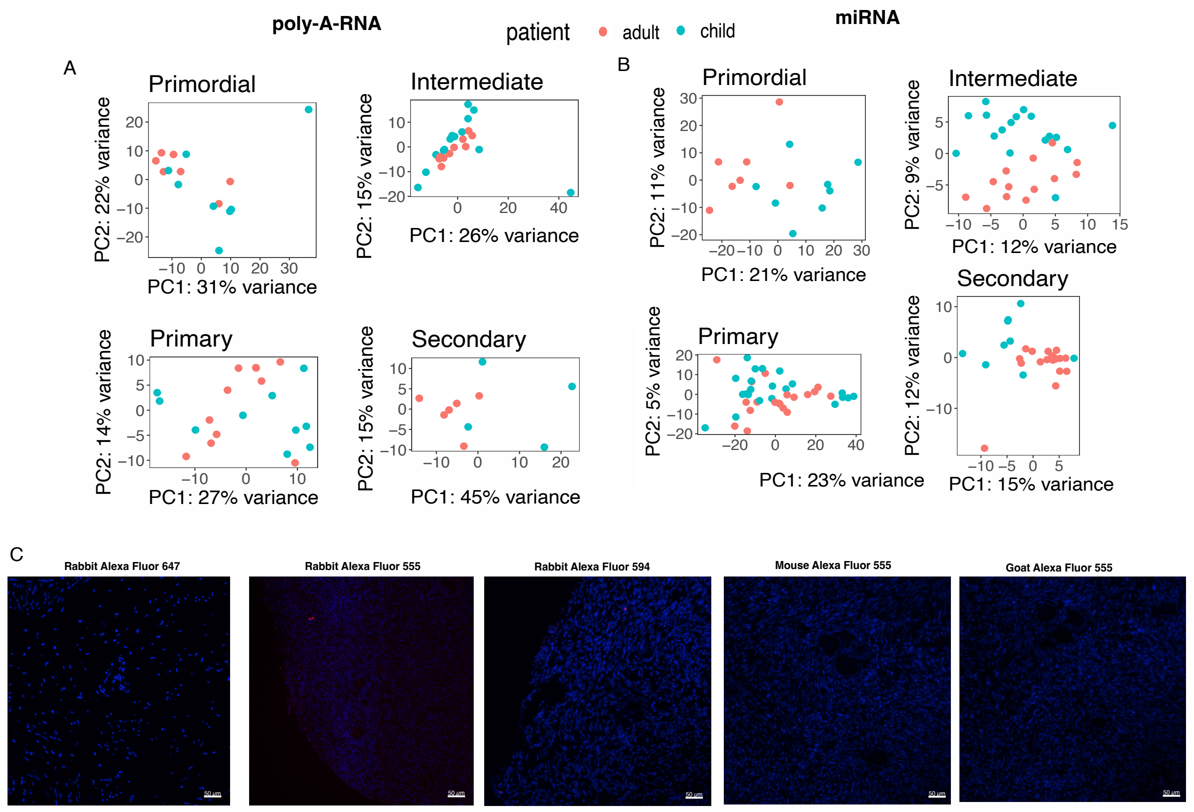

Supplementary Figure 5. Transcriptomic variations between child and adult follicles.
Child and adult follicles were categorized by morphologically determined growth stages to analyse transcriptomic differences.
A) Principal component analysis of Smart-seq2 results from Group 1 follicles (n=70 follicles) demonstrated separation between the age groups, with PC 1 notably separating the ages at the secondary stage.
B) A discernible separation was observed in the follicles based on their miRNA signatures too (n=113 follicles).
C) Negative controls for secondary antibodies.

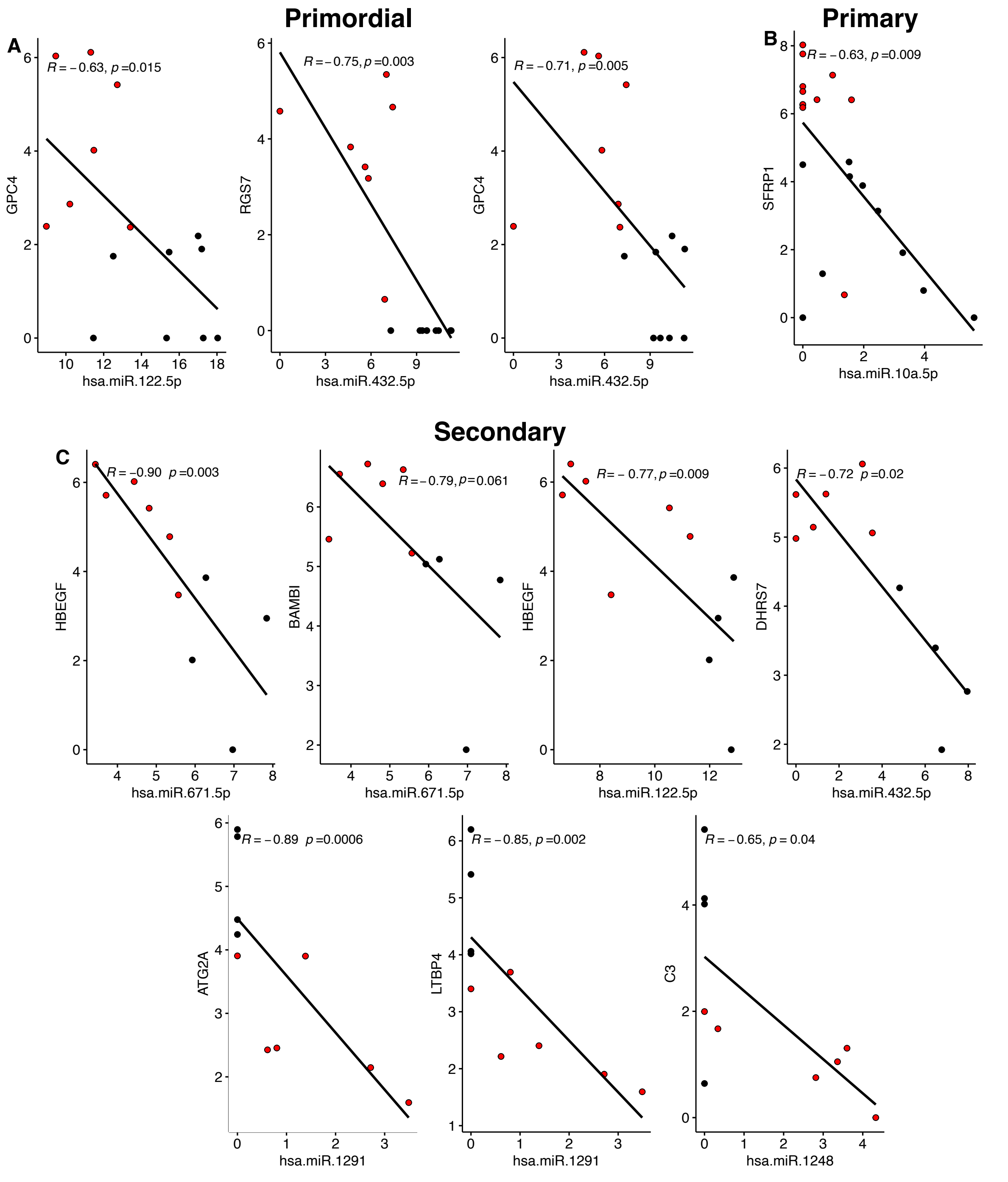

Supplementary Figure 6. Correlations between differentially expressed miRNAs and their predicted targets

The expression of miRNAs that were differentially expressed between child and adult follicles at the same stage was compared to their predicted targets using Spearman correlations. The analyses focused on those predicted mRNA targets that were identified as differentially expressed. Each dots represents a follicle. Correlation coefficients and their adjusted significance levels are indicated in the figures. Selected significant correlations are shown with roles in steroid hormone synthesis and metabolism (*DHRS7, HB-EFG,* and *BAMBI*), autophagy

and apoptosis (*ATAG2* and *LTBP4*), inflammation (*C3*), signaling (*SFRP1* and *RGS7*) and insulin sensitivity (*GPC4*).
A) Correlations from primordial follicles (adult n=7 and child n=8).
B) Correlation from primary follicles (adult n=11 and child n=10).
C) Correlations from secondary follicles (adult n=6 and child n=4).
Adult follicles are coloured red and child black.
FDR cut-off for mRNA <0.05 and for miRNA FDR <0.05 and fold change >1.5.

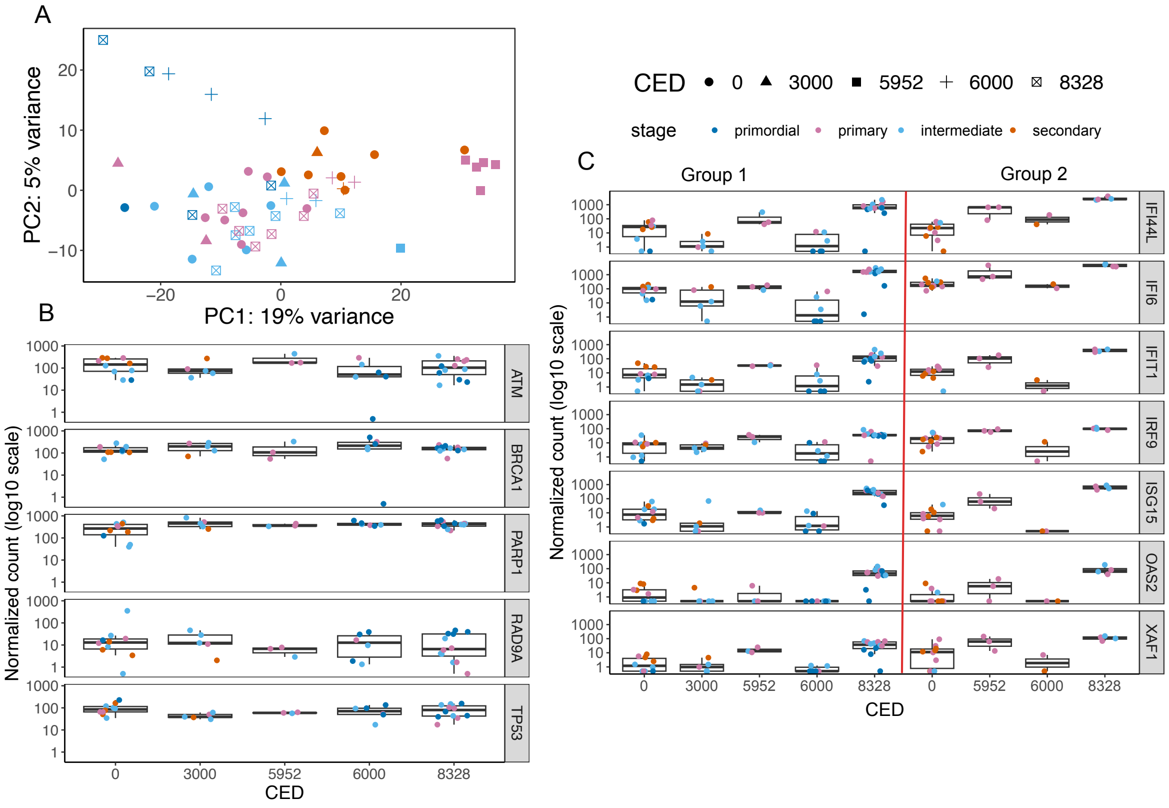

Supplementary Figure 7. Impact of chemotherapy on microRNA and gene expression in child follicles.

This figure illustrates the effect of chemotherapy on microRNA and gene expression within child follicles. The analysis reveals no discernible gradient in follicle clustering based on microRNA expression in relation to the cumulative dose of alkylating chemotherapy (CED, cyclophosphamide equivalent dose). Interestingly, no significant differences in the expression levels of DNA damage response genes were observed across varying CED doses. Additionally, both Group 1 and Group 2 follicles exhibited similar interferon gene expression following exposure to CED.

A) Principal component analysis depicting the clustering of child follicles based on microRNA expression. Samples are differentiated by their levels of CED exposure.

B) Assessment of DNA damage response gene expression in follicles subjected to different CED levels. In box plots, the center line represents the median, the hinges correspond to the first and third quartiles (interquartile range), and the whiskers extend to 1.5 times the interquartile range from the hinges.

C) Comparison of interferon signaling pathway gene expression between Group 1 and Group 2 follicles. In box plots, the center line represents the median, the hinges correspond to the first and third quartiles (interquartile range), and the whiskers extend to 1.5 times the interquartile range from the hinges.
