# Supplementary material for: In-depth analysis of transcriptomes in ovarian cortical follicles from children and adults reveals interfollicular heterogeneity
Source: Nat Commun. 2024 Aug 21;15:6989. doi: 10.1038/s41467-024-51185-0 (PMC11339373; doi:10.1038/s41467-024-51185-0)
Supplement: Supplementary file 3 — Description of additional supplementary files [file 41467_2024_51185_MOESM3_ESM.pdf]

Description of Additional Supplementary Files

Supplementary Data 1- qPCR analysis samples. Independent validation set of follicles after outlier removal. Immunofluorescence and RNA FISH validation samples and antibodies used for immunofluorescence staining. Related to Figure 4-6. and Supplementary Figure 1-2.

Supplementary Data 2- Functional enrichment analysis between two groups of follicles. Genes upregulated in Group 1 primary follicles. Genes upregulated in Group 2 primary follicles. Genes upregulated in Group 1 secondary follicles. Genes upregulated in Group 2 secondary follicles. Genes upregulated in Group 1 follicles (all stages combined). Genes upregulated in Group 2 follicles (all stages combined). For functional enrichment cut-off for differentially expressed genes FDR <0.05 and log2 fold change >1.5. Functional enrichment statistics are calculated using Fisher's one-tailed test and adjusted for multiple comparisons using the Benjamini-Hochberg method. Lists of differentially expressed genes between Group 1 and Group 2 follicles. Differentially expressed genes between all Group 1 and Group 2 primary follicles. Differentially expressed genes between Group 1 and Group 2 secondary follicles. Differentially expressed genes between all Group 1 and Group 2 follicles. Gene expression statistics are calculated using the Wald test and adjusted for multiple comparisons using the Benjamini-Hochberg method. Related to Figure 2 and Supplementary Figure 1.

Supplementary Data 3- Follicle distribution between patients and follicle stages in Group 1 (Smart-seq2). Follicle distribution between patients and follicle stages after outlier removal (small RNA sequencing). Related to Figures 3-4 and Supplementary Figure 5.

Supplementary Data 4- Gene expression patterns in ovarian follicles during the growth. Differentially expressed genes between different follicle stages in adult and child samples. The effect of follicle development is controlled by patient variables. Gene expression patterns in ovarian follicles during the growth. For pattern analysis differentially expressed genes from

different stage-wise comparisons were pooled. Gene expression statistics are calculated using the Wald test and adjusted for multiple comparisons using the Benjamini-Hochberg method. Gene was considered as significant if FDR <0.05. Related to Figure 3 and Supplementary Figure 4.

Supplementary Data 5- Functional enrichment analysis of gene patterns through ovarian follicle development and over-represenation analysis of microRNAs through ovarian follicle development. Genes direction up: Pattern 2 in adult and Pattern 1 in child. Genes direction down: Pattern 6 in adult and Pattern 3 in child. Adult diverse pattern: Pattern 20. Child diverse pattern: Pattern 10. Functional enrichment statistics are calculated using Fisher's one-tailed test and adjusted for multiple comparisons using the Benjamini-Hochberg method. Overrepresentation analysis of differentially expressed microRNAs in adult follicles. Overrepresentation analysis of differentially expressed microRNAs in child follicles. Overrepresentation statistics are calculated using Fisher's exact two-tailed test and adjusted for multiple comparisons using the Benjamini-Hochberg method.Related to Figure 3 and Supplementary Figure 4.

Supplementary Data 6- Differentially expressed genes between adult and child follicles between primordial follicles, intermediate follicles, primary follicles and secondary follicles. Gene expression statistics are calculated using the Wald test and adjusted for multiple comparisons using the Benjamini-Hochberg method. Gene was considered as significant if FDR <0.05. To assess the robustness of the differential expression analysis results, a bootstrapping analysis was conducted. The frequency column displays the number of times a gene was detected as differentially expressed across the 200 bootstraps. Related to Figure 4.

Supplementary Data 7- Functional enrichment analysis of differentially expressed genes between adult and child follicles (upregulated in adult follicles and upregulated in child follicles). The cut-off for differentially expressed genes FDR <0.05 and log2 fold change >1.5. Functional enrichment statistics are calculated using Fisher's one-tailed test and adjusted for multiple comparisons using the Benjamini-Hochberg method. Related to Figure Figure 4.

Supplementary Data 8- Differentially expressed microRNAs between adults and children within the same follicular stage (primordial follicles, primary follicles, secondary follicles). Gene expression statistics are calculated using the Wald test and adjusted for multiple comparisons using the Benjamini-Hochberg method. Gene was considered as significant if FDR <0.05.Related to Figure 4.

Supplementary Data 9- Spearman correlations between adult versus child differentially expressed genes (FDR <0.05) and differentially expressed microRNAs (FDR <0.05 and log2 fold change >1.5). Correlation statistics are calculated using a two-sided Spearman correlation test and adjusted for multiple comparisons using the Benjamini-Hochberg method. Related to Supplementary Figure 6.

Supplementary Data 10- Functional enrichment of microRNA target genes that displayed negative correlation with microRNA expression levels (primordial follicles and secondary follicles). Functional enrichment statistics are calculated using Fisher's one-tailed test and adjusted for multiple comparisons using the Benjamini-Hochberg method. Related to Figure 4.

Supplementary Data 11- Differentially expressed genes between activated and dormant primordial follicles. Gene expression statistics are calculated using the Wald test and adjusted for multiple comparisons using the Benjamini-Hochberg method. Gene was considered as significant if FDR <0.05. Related to Figure 5.
