# Supplementary material for: In-depth analysis of transcriptomes in ovarian cortical follicles from children and adults reveals interfollicular heterogeneity
Source: Nat Commun. 2024 Aug 21;15:6989. doi: 10.1038/s41467-024-51185-0 (PMC11339373; doi:10.1038/s41467-024-51185-0)
Supplement: Supplementary file 5 — Reporting Summary [file 41467_2024_51185_MOESM5_ESM.pdf]

nature portfolio

Corresponding author(s): Pauliina Damdimopoulou and Ilmatar Rooda

Last updated by author(s): Jun 26, 2024

Reporting Summary

Nature Portfolio wishes to improve the reproducibility of the work that we publish. This form provides structure for consistency and transparency in reporting. For further information on Nature Portfolio policies, see our Editorial Policies and the Editorial Policy Checklist.

Statistics

For all statistical analyses, confirm that the following items are present in the figure legend, table legend, main text, or Methods section.

n/a	Confirmed	
☐	☒	The exact sample size (*n*) for each experimental group/condition, given as a discrete number and unit of measurement
☐	☒	A statement on whether measurements were taken from distinct samples or whether the same sample was measured repeatedly
☐	☒	The statistical test(s) used AND whether they are one- or two-sided<br>*Only common tests should be described solely by name; describe more complex techniques in the Methods section.*
☐	☒	A description of all covariates tested
☐	☒	A description of any assumptions or corrections, such as tests of normality and adjustment for multiple comparisons
☐	☒	A full description of the statistical parameters including central tendency (e.g. means) or other basic estimates (e.g. regression coefficient) AND variation (e.g. standard deviation) or associated estimates of uncertainty (e.g. confidence intervals)
☐	☒	For null hypothesis testing, the test statistic (e.g. *F*, *t*, *r*) with confidence intervals, effect sizes, degrees of freedom and *P* value noted<br>*Give P values as exact values whenever suitable.*
☒	☐	For Bayesian analysis, information on the choice of priors and Markov chain Monte Carlo settings
☒	☐	For hierarchical and complex designs, identification of the appropriate level for tests and full reporting of outcomes
☐	☒	Estimates of effect sizes (e.g. Cohen's *d*, Pearson's *r*), indicating how they were calculated

Our web collection on statistics for biologists contains articles on many of the points above.

Software and code

Policy information about availability of computer code

Data collection	Image acquisition software: Nikon. qPCR: Roche LightCycler 480, Sequencing software: NextSeq500 and NCS version: 2.2.0.4
Data analysis	Images were processed using FIJI software (version 2.14.0, ImageJ2), OMERO and CellProfiler v4.2.5. InkScape software was used for image assembly.<br>Smart-seq2 analysis: Trimmomatic v 0.39 for quality filtering, STAR aligner v.2.7.1 for aligment and HTSeq v 0.11.2, Python v 3.6.4 for counting.<br>Small RNA data was analyzed: Trimmomatic v 0.39 for quality filtering, miRDeep2 for alignment and counting.<br>Downstream analyses for both Smart-seq2 and small RNA sequencing were performed using R with following packages:edgeR package v3.36.0 , DeSeq2 v.1.34.0 , umap v.0.2.9.0, DEGreport v.1.30.3, ggplot2 v.3.4.0, pheatmap v.1.0.12, EnhancedVolcano v.1.12.0, CellChat v.1.6.1. and Hmisc v.4.7-2. In addtion, online tools g:Profiler, ShinyGO and miEAA were used. No custom codes or algorithms were used to generate results.<br><br>Data analysis was performed in two age groups (adult and child). All patients were of the same sex, therefore eliminating age as a variable. Therefore, controlling for additional covariates was not applicable to this study because the primary factors of variability (age and sex) were already accounted for by the study design.

For manuscripts utilizing custom algorithms or software that are central to the research but not yet described in published literature, software must be made available to editors and reviewers. We strongly encourage code deposition in a community repository (e.g. GitHub). See the Nature Portfolio guidelines for submitting code & software for further information.

Data

Policy information about availability of data

All manuscripts must include a data availability statement. This statement should provide the following information, where applicable:

- Accession codes, unique identifiers, or web links for publicly available datasets
- A description of any restrictions on data availability
- For clinical datasets or third party data, please ensure that the statement adheres to our policy

Sequencing data is available in Gene Expression Omnibus (GEO) repository under the accession numbers GSE241982, GSE241981, GSE241983, GSE241984.

Research involving human participants, their data, or biological material

Policy information about studies with human participants or human data. See also policy information about sex, gender (identity/presentation), and sexual orientation and race, ethnicity and racism.

Reporting on sex and gender

Aim of the study was to analyze human ovarian follicle development and gene expression. Therefore, only female sex participants were recruited. Sex of the participants were determined by clinicians. Informed consent was obtained from all participants.

Reporting on race, ethnicity, or other socially relevant groupings

No race, ethnicity or social grouping information were collected during the study.

Population characteristics

Information about the participants age, diagnosis and treatments were collected.

Recruitment

Hospital personnel informed gender reassignment patients about the study and the patients who signed the informed consent were included. Childhood cancer patients who were at high or very high risk of iatrogenic premature ovarian insufficiency due to planned treatments were offered ovarian tissue cryopreservation (OTC). The provision of OTC was part of a research protocol in children. Written informed consent was obtained from all age-appropriate patients or guardians, and parental written consent was obtained for all patients under 18 years old.

Ethics oversight

For adult participants: Stockholm Region Ethical Review Authority (license number 2015/798-31 with amendments) and the Ethics Committee of Helsinki University Hospital (HUS/3319/2017 and HUS/2087/2023). Pediatric participants: Swedish Ethical Review Authority (Sveafertil patients, Dnr:2019-03802) and the Ethics Committee of Helsinki University Hospital (HCH patients, Dnr:340/13/03/03/2015).

Note that full information on the approval of the study protocol must also be provided in the manuscript.

Field-specific reporting

Please select the one below that is the best fit for your research. If you are not sure, read the appropriate sections before making your selection.

☒ Life sciences ☐ Behavioural & social sciences ☐ Ecological, evolutionary & environmental sciences

For a reference copy of the document with all sections, see nature.com/documents/nr-reporting-summary-flat.pdf

Life sciences study design

All studies must disclose on these points even when the disclosure is negative.

Sample size

No statistical method was used to predetermine the sample size. 120 ovarian follicles were obtained from n=9 individuals and used for Smart-seq2 and small RNA sequencing. Separate samples from 46 follicles collected from n=4 individuals were used for Smart-seq2 analysis. For immunofluorescence validation different set of individuals were used n=12.

The sample size of 120 follicles was chosen to ensure a sufficient number of observations for robust statistical analysis and to capture variability within and between the age groups. This number was considered sufficient to detect meaningful differences in gene expression patterns related to age. The inclusion of additional patients for validation was to confirm the findings from the primary analysis. This step was crucial to ensure the reproducibility and reliability of the results across a broader samples. By including multiple patients and a large number of follicles, we aimed to account for biological variability and increase the generalizability of our findings.

Data exclusions

From sequencing analysis outlier samples were excluded. Sample was considered as outlier if sample library size was considerably lower than remaining samples and plotted separately on the PCA analysis. In addition, two samples were excluded due to the low quality microscope image which did not allow to confirm sample stage.

Replication

Samples were collected and processed in two university laboratories yielding similar results, Smartseq-2 library preparation was performed in two different laboratories by two different researcher yielding with similar results. In addition for validation separate set of individuals were used to confirm the results.

Child ovarian samples were obtained from patients from fertility preservation program. Therefore, there is a potential self-selection bias in

child group. Technical bias may occur due to variations in sample handling, sequencing, and data processing. While potential biases exist, efforts were made to mitigate their impact through careful study design and standardized technical procedures.

Randomization

Human samples were used without randomization. Patients undergoing gender reassignment surgery, planned c-section or fertility preservation were selected to the study.
The study population was carefully selected to include patients from two critical age ranges, representing prepubescent children (1.5-11 years) and adults (19-36). This selection allowed for a focused investigation of ovarian follicle development and gene expression at different life stages, providing valuable insights into the biological processes underlying fertility and reproductive health.

Blinding

Blinding was not used in this study. Groups were based on the individuals age which was necessary for the hypothesis testing.

Reporting for specific materials, systems and methods

We require information from authors about some types of materials, experimental systems and methods used in many studies. Here, indicate whether each material, system or method listed is relevant to your study. If you are not sure if a list item applies to your research, read the appropriate section before selecting a response.

Materials & experimental systems

n/a	Involved in the study	
☐	☒	Antibodies
☒	☐	Eukaryotic cell lines
☒	☐	Palaeontology and archaeology
☒	☐	Animals and other organisms
☒	☐	Clinical data
☒	☐	Dual use research of concern
☒	☐	Plants

Methods

n/a	Involved in the study	
☒	☐	ChIP-seq
☒	☐	Flow cytometry
☒	☐	MRI-based neuroimaging

Antibodies

Antibodies used

Primary andtibodies: Goat anti-DDX4 (AF2040) R&D systems, Rabbit anti-FIGLA (HPA071241) Atlas antibodies, Rabbit anti-LHX8 (HPA077810) Atlas antibodies, Mouse anti-ACTA2 (MAB1420) R&D systems, Mouse anti-ASTN1 (sc-514299) Santa Cruz, Goat anti-SPARCL1 (AF2728) R&D systems, Rabbit anti-HIF3A (HPA041141) Atlas antibodies, Rabbit anti-IFI6 (HPA005543) Atlas antibodies, Rabbit anti-IFI6 (HPA005543) Atlas antibodies, Rabbit anti-IFI44L (HPA060372) Atlas antibodies. Secondary antibodies: Alexa Fluor 555 Donkey anti-Rabbit (A31572) Invitrogen, Alexa Fluor 555 Donkey anti-Mouse(A31570) Invitrogen, Alexa Fluor 555 Donkey anti-Goat (A21432) Invitrogen, Alexa Fluor 594 Donkey anti-Rabbit (A21207) Invitrogen, Alexa Fluor 647 Donkey anti-Rabbit (A31573) Invitrogen

Validation

Commercially available antibodies have been validated by the manufacturer. Antibodies obtained from Human Protein Atlas (HPA) were validated by HPA
